# Prognostic Value of N-terminal Probrain Natriuretic Peptide for Patients with Acute Respiratory Distress Syndrome: A Systematic Review and Meta-Analysis

**DOI:** 10.1155/2020/3472615

**Published:** 2020-04-05

**Authors:** Qi Ni, Chaoqian Li, Hua Lin

**Affiliations:** ^1^Department of Scientific Research, The First Affiliated Hospital of Guangxi University of Traditional Chinese Medicine, 89-9 Dongge Road, Nanning, 530023 Guangxi, China; ^2^Department of Emergency, The First Affiliated Hospital of Guangxi Medical University, 6 Shuangyong Road, Nanning, 530021 Guangxi, China; ^3^Intensive Care Unit, The First Affiliated Hospital of Guangxi University of Traditional Chinese Medicine, 89-9 Dongge Road, Nanning, 530023 Guangxi, China

## Abstract

**Objectives:**

The mortality rate of patients with acute respiratory distress syndrome (ARDS) is high. Hence, it is crucial to identify a reliable biomarker with wide clinical applications for predicting the prognosis of patients with ARDS. This systematic review and meta-analysis was conducted to investigate the value of plasma N-terminal probrain natriuretic peptide (NT-proBNP) for predicting mortality in patients with ARDS.

**Methods:**

An electronic search of databases including PubMed, Web of Science, Cochrane Library, and Chinese National Knowledge Infrastructure was conducted up to May 31, 2019, without language restrictions. The quality of the included studies was evaluated using QUADAS-2. Data were extracted and analyzed to obtain pooled estimates of sensitivity, specificity, positive likelihood ratio, negative likelihood ratio, and diagnostic odds ratio. A forest graph was used to evaluate heterogeneity. Potential causes of heterogeneity were further explored by subgroup analysis based on the testing day, testing method, observation endpoint, or cut-off points. A summary receiver operating characteristic curve was drawn to obtain the pooled area under the curve.

**Results:**

A total of 7 studies involving 581 patients with ARDS were included. The pooled sensitivity, specificity, positive likelihood ratio, negative likelihood ratio, and diagnostic odds ratio were as follows: 0.79 (95% CI: 0.72–0.84), 0.79 (95% CI: 0.66–0.88), 3.68 (95% CI: 2.16–6.28), 0.27 (95% CI: 0.20–0.38), and 13.58 (95% CI: 6.17–29.90), respectively. The results of subgroup analysis showed that the testing day influenced the summary sensitivity and that the cut-off points influenced the summary sensitivity and specificity.

**Conclusion:**

Our results indicate that elevated plasma NT-proBNP levels have a moderate value for predicting the mortality of patients with ARDS.

## 1. Introduction

Acute respiratory distress syndrome (ARDS) is a serious clinical disease with a mortality rate higher than 40% [1–3]. Early prediction of poor prognosis in ARDS patients after intensive care unit (ICU) admission would enable physicians to implement more effective treatment strategies to improve survival. Previous studies have shown that several biomarkers such as plasma angiotensin peptides and interleukin-18 are associated with the prognosis of ARDS patients [4, 5], but these biomarkers are not widely used in clinical applications. Therefore, it is crucial to identify a reliable biomarker that can predict the prognosis of these patients in a clinical setting.

Brain natriuretic peptide (BNP) is a hormone synthesized and secreted by cardiomyocytes. BNP and its inactive cleavage product, N-terminal pro-BNP (NT-proBNP), are derived from the cleavage of BNP precursors. NT-proBNP has a longer half-life and more stable biological characteristics than BNP, making it more suitable for clinical application [6, 7]. Plasma NT-proBNP has been widely used as an important biomarker of increased left ventricular filling pressure and left ventricular dysfunction [8, 9]. However, heart failure is not the only cause of increased plasma NT-proBNP levels; noncardiac causes include sepsis and shock, which may lead to myocardial tension and elevate NT-proBNP levels. Sepsis is the most common etiology of ARDS [10]. NT-proBNP levels are increased in patients with ARDS, a condition caused by a combination of factors [11–16].

Elevated NT-proBNP at admission is an independent predictor of ICU outcomes [17, 18]. NT-proBNP has been identified as an effective predictor of death and major adverse cardiovascular events in patients with stable coronary disease [19] or in those who have undergone noncardiac surgery [20]. It was reported that for patients admitted to the ICU without decompensated heart failure or acute coronary syndrome, NT-proBNP concentrations were significantly elevated, particularly in patients with sepsis, and NT-proBNP strongly and independently predicted mortality [21]. Moreover, a previous study reported that NT-proBNP has a moderate predictive value for the mortality of patients with sepsis [22]. NT-proBNP has been shown to be more accurate than troponin T and troponin I for detecting mortality in ARDS [23]. Additionally, NT-proBNP concentrations are strongly associated with morbidity and mortality in patients with ARDS, with comparable predictive accuracy to more complex tools such as the Acute Physiology and Chronic Health Evaluation (APACHE) III score [24].

Some studies have investigated the potential prognostic value of plasma NT-proBNP for patients with ARDS [25–32]. However, the number of patients enrolled in these studies was limited and different testing times, methods, or clinical endpoints were used. A meta-analysis combines the results of multiple studies with statistical methods to provide more reliable results than individual studies. Here, we explored the value of plasma NT-proBNP in predicting the mortality of patients with ARDS.

## 2. Materials and Methods

### 2.1. Search Strategy

PubMed, Web of Science, Cochrane Library, and Chinese National Knowledge Infrastructure (up to May 31, 2019) were searched using the following keywords: “N-terminal pro-brain natriuretic peptide” or “NT-proBNP,” AND “acute respiratory distress syndrome” or “ARDS,” “acute lung injury” or “ALI.” No language restrictions were applied.

The inclusion criteria were as follows: (1) the included articles were limited to human studies; (2) the study evaluated the prognostic value of plasma NT-proBNP for patients with ARDS; (3) all patients met the criteria for ARDS and were administered standard treatment for ARDS; (4) the sensitivity and specificity of plasma NT-proBNP were provided; and (5) the outcomes (survival and nonsurvival) of patients and study endpoints were provided.

The exclusion criteria were as follows: (1) review articles, (2) conference abstracts, (3) animal studies, and (4) incomplete data.

### 2.2. Data Extraction and Quality Assessment

Two independent reviewers screened articles, extracted data, and identified relevant studies. Any disagreements were resolved by discussion. The following data were extracted: names of the first authors, publication year, country, number of patients, sampling timing, plasma NT-proBNP evaluation method, plasma NT-proBNP cut-off value, outcome assessment, area under the curve (AUC), NT-proBNP sensitivity and specificity values, and observation endpoint. True-positive (TP), false-positive (FP), false-negative (FN), and true-negative (TN) rates were calculated according to the following formulas: TP = the number of nonsurvival patients multiplied by sensitivity; FP = the number of survival patients multiplied by (1 − specificity); FN = the number of nonsurvival patients multiplied by (1 − sensitivity); and TN = the number of survival patients multiplied by specificity. Study quality was assessed using the revised tool-2 for Quality Assessment of Diagnostic Accuracy Studies (QUADAS-2) [33]. The evaluation of risk of bias and clinical applicability includes “low,” “high,” and “unclear.”.

### 2.3. Statistical Analysis

All analyses were performed using STATA 15 software (StataCorp LP, College Station, TX, USA), and *P* < 0.05 was considered statistically significant. The following summary measures were calculated: sensitivity and specificity, positive likelihood ratio (PLR), negative likelihood ratio (NLR), and diagnostic odds ratio (DOR) with corresponding 95% confidence intervals. Cochrane's *Q* test and the inconsistency index (*I*^2^) were used to assess the degree and significance of heterogeneity across eligible studies [34]. An *I*^2^ > 50% indicated significant heterogeneity. A random effects model was used to calculate parameters when there was significant heterogeneity; otherwise, a fixed effects model was used. A forest map was used to graphically depict the overall sensitivity and specificity as well as the heterogeneity across all included studies. PLR, NLR, and DOR were calculated according to the summary sensitivity and specificity. The SROC curve was used to obtain the pooled AUC. Heterogeneity among the results of different studies was explored by subgroup and metaregression analyses [22]. Publication bias was assessed using Deeks' test [35]. A *P* > 0.10 was considered to indicate a lack of publication bias.

## 3. Results

### 3.1. Summary of Eligible Studies

**Figure 1:**
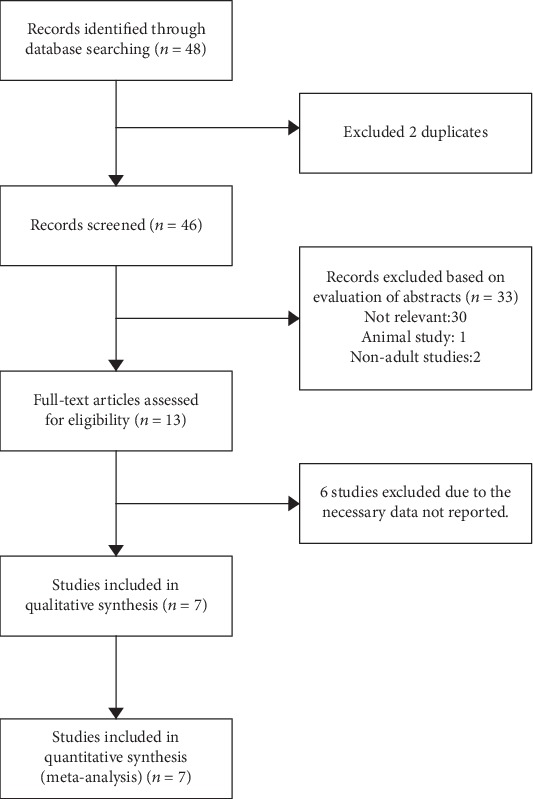
Flow diagram of the study selection process.

A flow chart of the literature screening process is shown in Figure 1. A total of 48 related publications were initially identified, and 2 duplicates were excluded. After screening the abstracts, 33 articles were excluded as they were either animal studies, nonadult studies, or irrelevant to this meta-analysis. Additional 6 of the remaining 13 articles were excluded after full-text screening as they did not provide data on the sensitivity and specificity of NT-proBNP. Finally, 7 studies [25–27, 29–32] involving 581 patients with ARDS were included in this meta-analysis.

### 3.2. Characteristics of Included Studies

The characteristics of the included studies are shown in Table 1. Plasma NT-proBNP levels were evaluated using an Elecsys 2010 analyzer (Roche, Basel, Switzerland) [25–27, 29, 31] or other methods [30, 32]. Five studies [26, 27, 29–31] used 28-day mortality as the endpoint, whereas 30-day [32] and 60-day [25] mortalities were each used as the endpoint in a single study.

### 3.3. Quality Assessment

Quality assessments of the included studies are presented in Table 2. The present meta-analysis indicates that the major risk of bias for the included studies occurred during index tests. The risk of bias for the index test in 5 studies was labeled high due to the fact that the threshold was not prespecified [25, 29–32]. The risk bias for the index test of one other study was labeled as unknown because it did not report whether or not the threshold was prespecified [27].

### 3.4. Prognostic Value of Plasma NT-proBNP for Patients with ARDS

**Figure 2:**
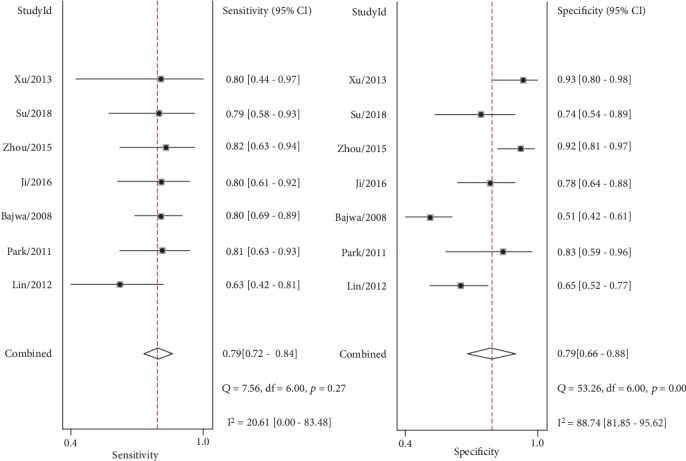
Summary sensitivity and specificity plotted on forest graphs for NT-proBNP in predicting the mortality of patients with ARDS.

**Figure 3:**
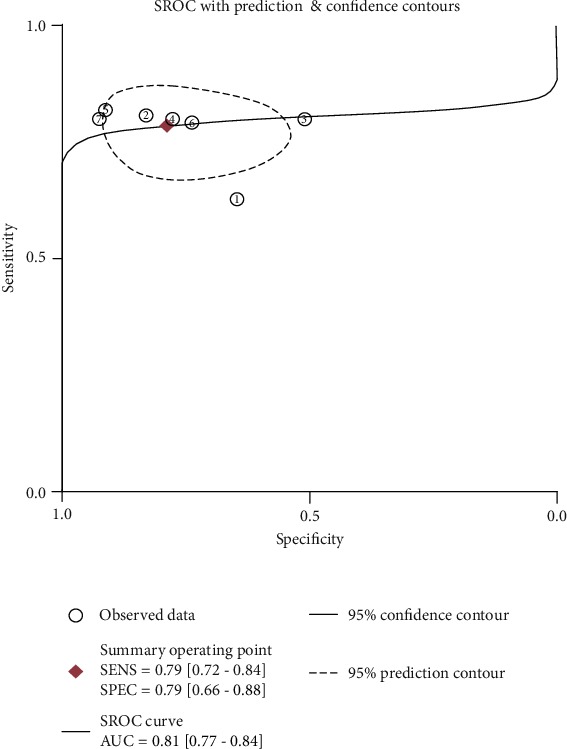
SROC curve for NT-proBNP in predicting the mortality of patients with ARDS.

The overall prognostic sensitivity, specificity, PLR, NLR, and DOR of plasma NT-proBNP were 0.79 (95% CI: 0.72–0.84), 0.79 (95% CI: 0.66–0.88), 3.68 (95% CI: 2.16–6.28), 0.27 (95% CI: 0.20–0.38), and 13.58 (95% CI: 6.17–29.90), respectively. Although no significant heterogeneity was observed among studies regarding combined sensitivity (*I*^2^ = 20.61%, *P* > 0.05), the heterogeneity of combined specificity among studies was significant (*I*^2^ = 88.74%, *P* < 0.01) (Figure 2). The pooled area under the receiver operating characteristic (ROC) curve (AUC) was 0.81 (95% CI 0.77–0.84) for serum NT-proBNP in the prognosis of ARDS (Figure 3).

### 3.5. Subgroup Analysis and Metaregression Analyses for Plasma NT-proBNP

The potential causes of heterogeneity were further explored by subgroup analysis based on the testing day, testing method, observation endpoint, or cut-off points (>1800 and <1800 pg/mL or no data provided). The results of subgroup analysis and meta-regression analyses showed that the summary sensitivity and specificity of plasma NT-proBNP in the testing method subgroup or observation endpoint subgroup did not significantly differ (*P* > 0.05). However, the summary sensitivity and specificity of the cut-off point subgroup were significantly different (*P* < 0.01). Although the summary sensitivity of plasma NT-proBNP tested at day one significantly differed compared to that on other days (*P* < 0.05), contrasting results were found in the subgroup analysis of the summary specificity (*P* > 0.05) (Table 3).

### 3.6. Publication Bias Analysis

**Figure 4:**
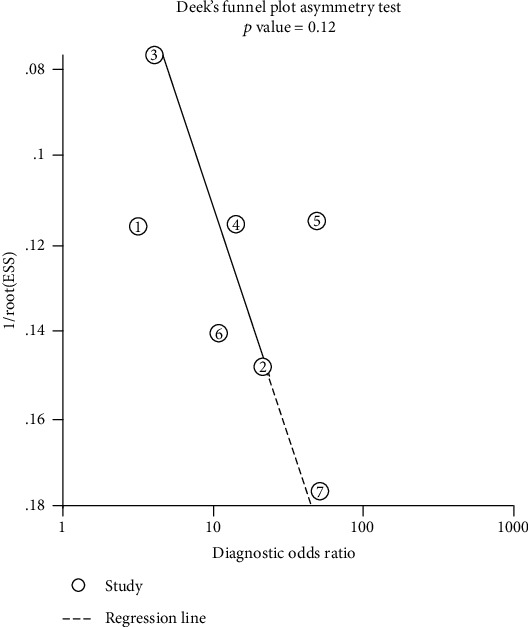
Deeks' funnel plot asymmetry test of NT-proBNP in predicting the mortality of patients with ARDS.

No publication bias was found for serum NT-proBNP in the prognosis of ARDS as assessed by Deeks' funnel plot (Deeks' test: *P* = 0.12; Figure 4).

## 4. Discussion

Seven relevant studies were identified for inclusion in this review. We evaluated the value of plasma NT-proBNP in predicting the mortality of patients with ARDS. Meta-analysis indicated that NT-proBNP has a moderate prognostic value for patients with ARDS. The overall sensitivity and specificity of plasma NT-proBNP for the prognosis of patients with ARDS were 0.79 and 0.79, respectively, and the AUC of SROC was 0.81, indicating that NT-proBNP appears to be a prognostic marker for ARDS. PLR and NLR directly reflect the clinical utility of an index test for a target disease.

As shown in Figure 2, the heterogeneity of the combined specificity across eligible studies was significant (*I*^2^ = 88.74%, *P* < 0.01); however, the heterogeneity of the combined sensitivity was not significant (*I*^2^ = 20.61%, *P* > 0.05). Thus, we performed subgroup analysis to investigate the sources of heterogeneity. The results in Table 3 indicate that differences neither in testing days and methods nor in observation endpoints significantly influenced the summary specificity (*P* > 0.05). However, the analysis of the subgroup cut-off points and meta-regression analyses indicated statistical significance for sensitivity and specificity (*P* < 0.01), indicating that different cut-off points influenced heterogeneity, especially in terms of specificity. Meanwhile, we were unable to determine an ideal cut-off value for the prognosis of ARDS patients as the cut-off value varied greatly across the included studies. No publication bias was found in the included studies based on Deeks' funnel plot, which demonstrates that the conclusions of this study are stable and reliable.

As a widely available biomarker in clinical practice, the measurement of NT-proBNP is convenient compared with other prognostic biomarkers. However, the increase of plasma NT-proBNP levels in patients with ARDS is caused by a combination of factors. In ARDS patients needing mechanical ventilation, NT-proBNP may be elevated because of the high intrathoracic pressure that develops during ventilation and the strain imposed on the right ventricle which faces a diseased and partially collapsed/embolized pulmonary vascular tree. In addition to increased ventricular filling pressure and ventricular dysfunction, endotoxin and inflammatory factors such as interleukin-6 increase plasma NT-proBNP levels by stimulating BNP gene expression in myocardial cells [13, 16]. Sympathetic excitation and use of vasoactive drugs may also directly stimulate cardiac myocytes to release NT-proBNP. Therefore, factors that significantly influence elevated plasma NT-proBNP should be comprehensively considered in clinical practice. In addition, if plasma NT-proBNP can be combined with other common indicators such as APACHE scores, the accuracy of ARDS patient prognosis will increase. A previous study has reported that BNP has a moderate prediction value for the mortality of septic patients [22] and BNP levels may be valuable in evaluating the prognosis of patients with ARDS [36, 37]. Unfortunately, the number of articles on BNP as a prognostic indicator in patients with ARDS is inadequate for meta-analysis at this time.

The major strength of our study is that we followed standard and up-to-date procedures to conduct this review and that we analyzed the prognostic value of NT-proBNP using the subgroup analysis of the testing day, testing method, observation endpoint, and cut-off points, which provided moderate reference information for future clinical applications. For example, plasma NT-proBNP levels of patients with ARDS should be tested early in the admission process to evaluate prognosis, which may help identify patients who would benefit from more active treatment strategies early on. Further studies with larger sample sizes should be conducted to determine the ideal cut-off value for clinical applications. There are some limitations to this meta-analysis worth noting. First, the number of eligible studies and sample size were relatively small, which might weaken the conclusion of this analysis. Second, all included studies were published in English or Chinese, leaving the possibility that studies in other languages and unpublished results may not have been included in this meta-analysis. Third, our pooled analysis did not take into account other potential confounding factors such as causes of ARDS and the gender and age of patients because we did not have access to the related raw data.

## 5. Conclusions

This systematic review and meta-analysis demonstrates that elevated plasma NT-proBNP appears to be a prognostic marker in ARDS. However, considering that there are many potential confounding factors in clinical treatment, more large-scale prospective studies should be conducted to more accurately assess the prognostic value of NT-proBNP in patients with ARDS.

## Figures and Tables

**Table 1:** Characteristics of included studies.

Author	Year	Country	Tested days	Cut-off	Survival/nonsurvival	AUC	Sensitivity (%)	Specificity (%)	Endpoint	Test method	TP	FP	FN	TN
Lin [32]	2012	China	d1	2417 pg/mL	60/27	0.722	61.5	65.6	30 d mortality	Roche, Cobas e411 analyzer	17	21	10	39
Park [26]	2011	Korea	d2	Percent change 30%	18/31	0.82	82	81	28 d mortality	Roche, Elecsys 2010 analyzer	25	3	6	15
Bajwa [25]	2008	America	d2	6813 ng/L	107/70	0.66	80	51	60 d mortality	Roche, Elecsys 2010 analyzer	56	52	14	55
Ji [29]	2016	China	d1	4522 ng/L	30/50	0.852	81	77	28 d mortality	Roche, Elecsys 2010 analyzer	24	11	6	39
Zhou [30]	2015	China	d1	333.92 pg/mL	59/28	0.798	81.92	91.89	28 d mortality	NA	23	5	5	54
Su [27]	2018	China	d1	NA	27/24	0.832	79.2	74.1	28 d mortality	Roche, Elecsys 2010 analyzer	19	7	5	20
Xu [31]	2013	China	d1	335 pg/mL	10/40	0.960	80.0	92.5	28 d mortality	Roche, Elecsys 2010 analyzer	8	3	2	37

NA: not available; AUC: area under the curve; TP: true positive; FP: false positive; TN: true negative; FN: false negative.

**Table 2:** Quality assessment of eligible studies.

Study	Risk of bias				Applicability concerns		
	Patient selection	Index test	Reference standard	Flow and timing	Patient selection	Index test	Reference standard
Lin et al. [32]	Low	High	Low	Low	Low	Low	Low
Park et al. [26]	High	Low	Low	Low	Low	Low	Low
Bajwa et al. [25]	Low	High	Low	High	Low	Low	Low
Ji et al. [29]	Low	High	Low	Low	Low	Low	Low
Zhou and Hua [30]	Low	High	Low	Low	Low	Unclear	Low
Su et al. [27]	Unclear	Unclear	Low	Low	Unclear	Low	Low
Xu et al. [31]	Low	High	Low	Low	Low	Low	Low

**Table 3:** Subgroup analysis and metaregression analyses of NT-proBNP.

	Subgroup	Studies (*n*)	Sensitivity	P1	Specificity	P2
Tested day	Day 1	5	0.77 [0.69-0.85]	0.01	0.82 [0.73-0.92]	0.59
	Non-day 1	2	0.81 [0.72-0.90]		0.65 [0.42-0.88]	

Tested method	Elecsys 2010 analyzer	5	0.81 [0.74-0.88]	0.30	0.77 [0.64-0.91]	0.41
	Non-Elecsys 2010 analyzer	2	0.73 [0.60-0.85]		0.81 [0.63-0.99]	

Endpoint	28 d mortality	5	0.80 [0.73-0.88]	0.11	0.85 [0.79-0.91]	0.41
	Non-28d mortality	2	0.74 [0.65-0.84]		0.58 [0.46-0.69]	

Cut-off points	>1800 pg/mL	3	0.76 [0.68-0.84]	0.00	0.64 [0.52-0.77]	0.00
	<1800 pg/mL or no data provided	4	0.81 [0.73-0.89]		0.87 [0.80-0.94]	
